# *CpWRKY71*, a WRKY Transcription Factor Gene of Wintersweet (*Chimonanthus praecox*), Promotes Flowering and Leaf Senescence in *Arabidopsis*

**DOI:** 10.3390/ijms20215325

**Published:** 2019-10-25

**Authors:** Renwei Huang, Daofeng Liu, Min Huang, Jing Ma, Zhineng Li, Mingyang Li, Shunzhao Sui

**Affiliations:** Chongqing Engineering Research Center for Floriculture, Key Laboratory of Horticulture Science for Southern Mountainous Regions of Ministry of Education, College of Horticulture and Landscape Architecture, Southwest University, Chongqing 400715, China; swuhrw@126.com (R.H.); liu19830222@163.com (D.L.); yellowrose11@163.com (M.H.); majing427@swu.edu.cn (J.M.); znli@swu.edu.cn (Z.L.)

**Keywords:** wintersweet, WRKY, expression pattern, promoter, flowering, senescence

## Abstract

The WRKY transcription factors are one of the most important plant-specific transcription factors and play vital roles in various biological processes. However, the functions of *WRKY* genes in wintersweet (*Chimonanthus praecox*) are still unknown. In this report, a group IIc *WRKY* gene, *CpWRKY71*, was isolated from wintersweet. CpWRKY71 was localized to the nucleus and possessed transcriptional activation activity. qRT-PCR (quantitative real-time PCR) analysis showed that *CpWRKY71* was expressed in all tissues tested, with higher expression in flowers and senescing leaves. During the flower development, the highest expression was detected in the early-withering stage, an obvious expression of *CpWRKY71* was also observed in the flower primordia differentiation and the bloom stage. Meanwhile, the expression of *CpWRKY71* was influenced by various abiotic stress and hormone treatments. The expression patterns of the *CpWRKY71* gene were further confirmed in *CpWRKY71pro*:*GUS* (*β-glucuronidase*) plants. Heterologous overexpression of *CpWRKY71* in *Arabidopsis* caused early flowering. Consistent with the early flowering phenotype, the expression of floral pathway integrators and floral meristem identity (FMI) genes were significantly up-regulated in transgenic plants. In addition, we also observed that the transgenic plants of *CpWRKY71* exhibited precocious leaf senescence. In conclusion, our results suggested that *CpWRKY71* may be involved in the regulation of flowering and leaf senescence in *Arabidopsis*. Our study provides a foundation for further characterization of *CpWRKY* genes function in wintersweet, and also enrich our knowledge of molecular mechanism about flowering and senescence in wintersweet.

## 1. Introduction

Wintersweet (*Chimonanthus praecox*), a perennial deciduous shrub of the *Calycanthaceae*, is native to China and widely distributing in south-central and southwest China [1]. Wintersweet, with its beautiful color, sweet fragrance, and long flowering period (from November to March), serves as an important winter-season ornamental tree in China and is widely used as landscape plants, pot plants and cut flowers [2]. Due to its unique flowering time, some researchers have paid attention to wintersweet flower development. To date, some progress has been made in this area. For instance, the wintersweet *APETALA3* (*CpAP3*) gene was found to partially rescue stamen development in the *Arabidopsis ap3* mutant, and caused numerous morphological changes and homeotic conversions to flowers [3]. Wintersweet *AGAMOUS-like6* (*CpAGL6*) gene was reported to promote flowering through inhibition of *FLOWERING LOCUS C* (*FLC*) and activation of *APETALA1* (*AP1*) and *FLOWERING LOCUS T* (*FT*) and cause abnormal stamen and carpel development in later developing flowers [4]. *CpCZF1* and *CpCZF2*, two C3H type zinc finger protein genes of wintersweet, were revealed to affect stamen development and caused the formation of abnormal flowers in transgenic *Arabidopsis* plants [5]. Additionally, for ornamental plants and cut flowers, the research on senescence is always one of the hot issues, which is closely related to its commercial value and market competitiveness [6]. In recent years, Sui et al. [7] reported the effects of hormones on the wintersweet cut flower senescence. However, little information is available regarding the molecular mechanisms of wintersweet senescence. According to previous research, plant growth and development processes, such as seed germination, secondary wall formation, flowering, and senescence, are regulated by a complex genetic network, in which transcriptional modulation is a crucial aspect of the complex genetic networks [8,9,10].

In the past decades, a growing number of transcription factor genes have been isolated and proved to widely participate in various developmental and physiological processes. Among them, the WRKY transcription factor family is one of the largest plant-specific transcription factor families. They are named after the conserved motif WRKYGQK and contain one or two conserved WRKY domains which are comprised of 60 amino acids at the N-terminal end and a zinc finger-like motif either C2H2 or C2HC at the C-terminal. Based on the number of WRKY domains and the type of zinc-finger motif, the family can be classified into three subfamilies, namely, group І (two WRKY domains and C2H2 zinc-finger), group ІІ (one WRKY domain and C2H2 zinc-finger) and group Ш (one WRKY domain and C2HC zinc-finger). Additionally, based on additional structural motifs outside the WRKY domain, group ІІ can be further divided into five subgroups (IIa, IIb, IIc, IId, and IIe). WRKY proteins regulate the expression of downstream genes by recognizing and binding to the W-box (TTGACC/T) present in their promoters [11]. Since *SPF1* (the first *WRKY* gene) was isolated from *sweet potato* [12], a large number of *WRKY* genes have been found subsequently in various plants [13,14,15,16,17,18]. According to previous reports, WRKY proteins are widely involved in plant developmental and physiological processes, and various biotic and abiotic stress responses. For example, *Arabidopsis* WRKY41 was reported to control *Arabidopsis* seed dormancy by directly regulating the expression of *ABI3* [19]. Overexpression of cotton *GhWRKY17* accelerated leaf senescence in *A. thaliana* [20]. *Dlf1*, a *WRKY* transcription factor gene which was isolated from rice, was reported to participate in the regulation of flowering time and plant height in rice [21]. Overexpression of Chrysanthemum *CmWRKY48* enhanced aphid resistance of transgenic chrysanthemum [22]. Overexpression of wheat *TaWRKY93* in *A. thaliana* enhanced multiple abiotic stress tolerances [23].

At present, the function of WRKY transcription factor has been well elucidated in model plants. However, to some extent, this family has not been well studied in some non-model plants or woody plants. To our knowledge, no information is available about the isolation and functional analysis of *WRKY* genes in wintersweet. We noted a novel *WRKY* gene, *CpWRKY71*, appeared to be differentially expressed during flower development, based on the information from the wintersweet transcriptome database [24]. Here, we isolated and characterized a novel *WRKY* gene *CpWRKY71* from wintersweet. The expression pattern of *CpWRKY71* was investigated. Ectopic expression of *CpWRKY71* in *Arabidopsis* resulted in an early flowering and precocious leaf senescence phenotype.

## 2. Results

### 2.1. Isolation and Characterization of CpWRKY71

**Figure 1:**
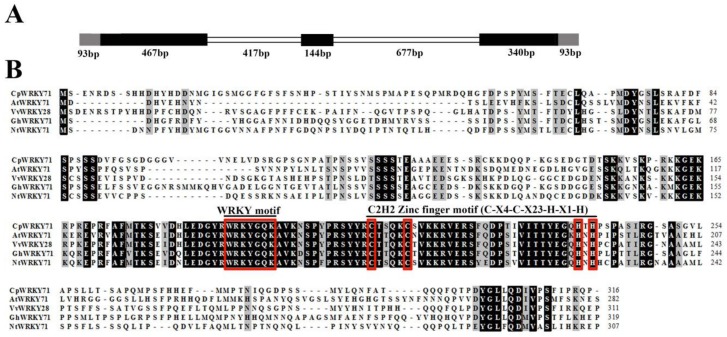
Sequence analysis of *CpWRKY71*. (**A**) Schematic diagram of *CpWRKY71* gene. Exons are indicated by black areas, introns are indicated by white areas, and untranslated regions are indicated by gray areas. (**B**) Multiple alignment of CpWRKY71 and other WRKY proteins from different plant species. *Arabidopsis thaliana* AtWRKY71(AEE31143), *Vitis vinifera* VvWRKY28 (RVW39625), *Gossypium hirsutum* GhWRKY71 (NP_001313893), and *Nicotiana tomentosiformis* NtWRKY71 (XP_009615724) are from GenBank. Identical and similar amino acids were shaded in black and gray, respectively. The conserved WRKY motif and zinc finger motif are marked by the red box.

**Figure 2:**
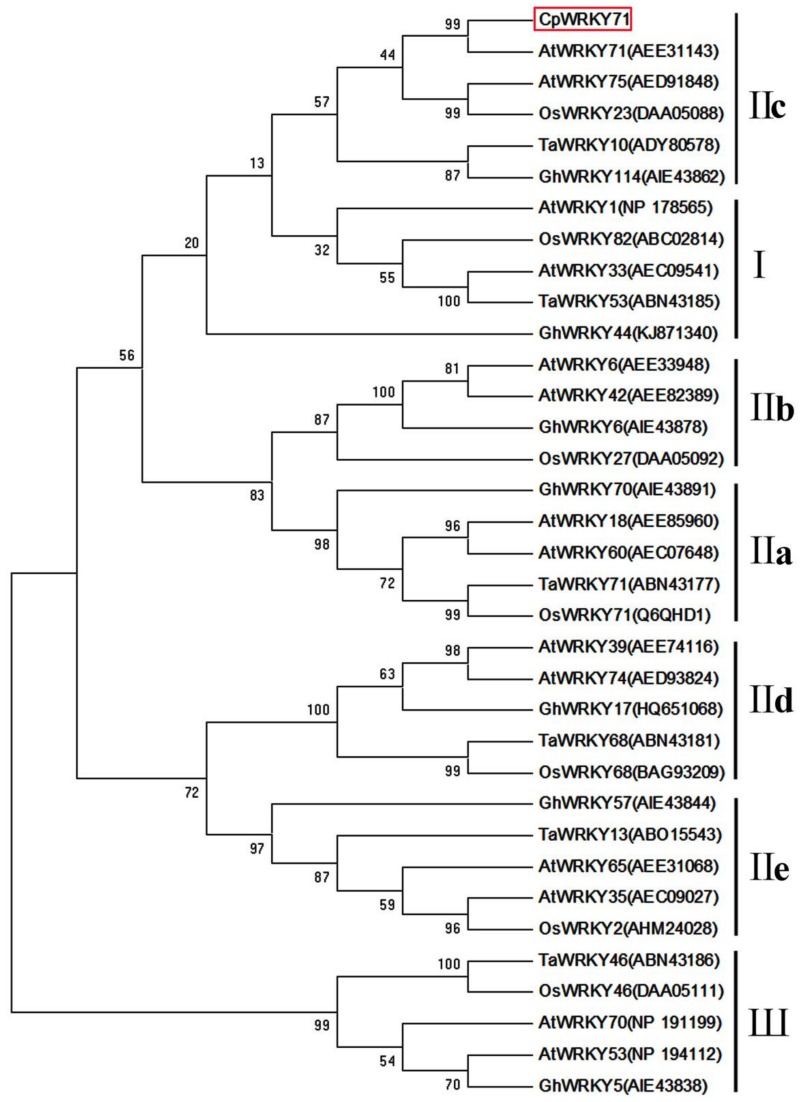
Phylogenetic tree of CpWRKY71 and WRKY proteins from other plant species. Phylogenetic analysis was performed by the neighbor-joining (NJ) method with 1000 bootstrap replicates using MEGA 5.0. At, *Arabidopsis thaliana*; Gh, *Gossypium hirsutum*; Ta, *Triticum aestivum*; and Os, *Oryza sativa*. GenBank accession numbers are in the brackets following the protein names. CpWRKY71 is marked by the red box.

Based on the sequence from the wintersweet flower transcriptome database, a 1137 bp full-length cDNA of *CpWRKY71* was obtained. Sequence analysis showed that *CpWRKY71* contained a complete open reading frame of 951 bp, which encodes a protein of 316 amino acids. Its predicted molecular weight and theoretical isoelectric point were 34.96 kDa and 6.72, respectively. Subsequently, we isolated 2231 bp genomic DNA fragment of *CpWRKY71*. Compared with cDNA sequence of *CpWRKY71*, the genomic DNA of *CpWRKY71* contained two introns (417 bp and 677 bp) and three exons (467 bp, 144 bp, and 340 bp) (Figure 1A). Sequence alignments indicated that CpWRKY71 showed high similarity with its homologous sequences, including AtWRKY71(AEE31143), VvWRKY28 (RVW39625), GhWRKY71 (NP_001313893) and NtWRKY71 (XP_009615724). CpWRKY71 contains a single WRKY domain and a C2H2-type zinc finger motif (Figure 1B), indicating that this protein belongs to group II WRKY family. The phylogenetic tree of CpWRKY71 and other WRKY proteins was constructed by MEGA 5.0. The results show that these WRKY proteins can be classified into three groups. CpWRKY71 was clustered with IIc subgroup proteins, including AtWRKY71, AtWRKY75, OsWRKY23, TaWRKY10, GhWRKY114, and was most closely related to AtWRKY71 (Figure 2), so we designated it as *CpWRKY71*. These results suggest that CpWRKY71 belongs to Group IIc of the WRKY transcription factor family.

### 2.2. CpWRKY71 is a Nuclear Protein with Transcriptional Activation Activity in Yeast

**Figure 3:**
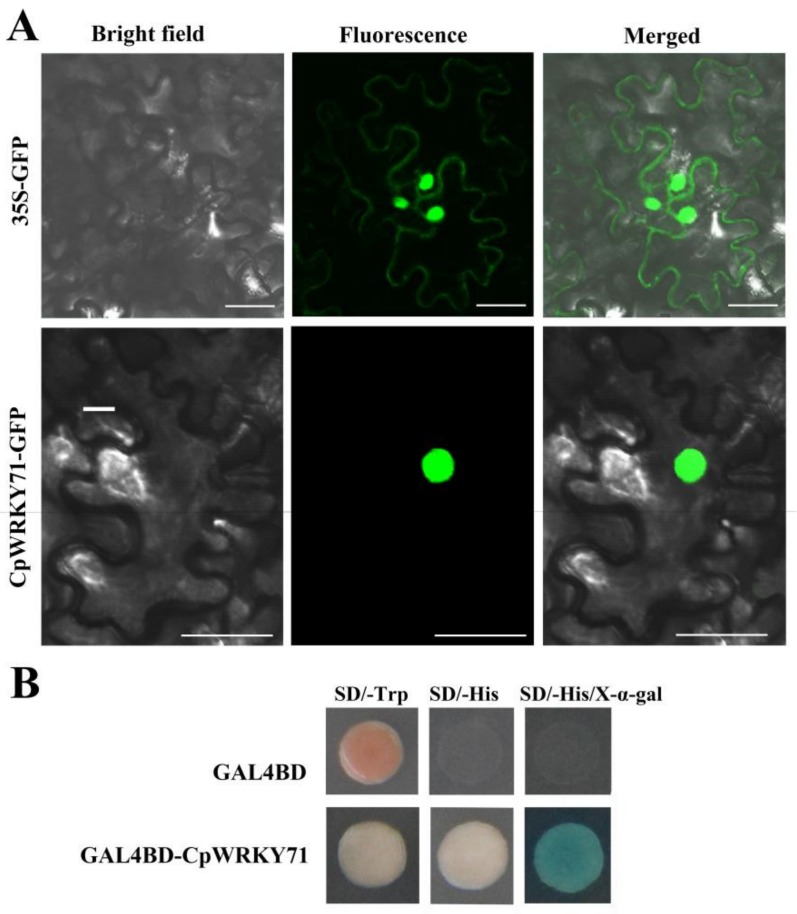
Subcellular localization and transactivation assay of CpWRKY71. (**A**) Subcellular localization of CpWRKY71 protein in tobacco leaf epidermal cells. 35S:GFP was used as the control. A confocal microscope was used to observe green fluorescence. Bars denote 50 µm. (**B**) Transactivation assay of CpWRKY71. The plasmid of pGBKT7-*CpWRKY71* was transformed into AH109 yeast strain, and examined on SD/Trp-, SD/-His (containing 10 mM 3-AT) and SD/-His/X-α-gal (containing 10 mM 3-AT) mediums. pGBKT7 was used as the control.

To investigate the subcellular localization of CpWRKY71, *CpWRKY71* was fused to the N-terminus of *GFP* (green fluorescent protein) gene. The *Agrobacterium tumefaciens* cells harboring *35S*:*CpWRKY71*-*GFP* or *35S*:*GFP* constructs were infiltrated to young leaves of tobacco (*Nicotiana benthamiana*) plants, respectively. The GFP fluorescence of CpWRKY71-GFP was observed in the nucleus, whereas that of GFP control was expressed in the cytoplasm and nucleus. These results indicate that CpWRKY71 is a nuclear-localized protein (Figure 3A).

Further, the yeast assay system was used to investigate whether CpWRKY71 has transcriptional activity. The plasmids pGBKT7 and pGBKT7-*CpWRKY71* were introduced into AH109 yeast strain, respectively. The results show that yeast cells harboring the plasmid pGBKT7-*CpWRKY71* could grow normally on SD/-His medium containing 10 mM 3-amino-1,2,4-triazole (3-AT), and show α-galactosidase activity on the plates containing X-α-gal. On the contrary, the yeast cells transfected with pGBKT7 could survive on the SD/-Trp medium only. This result indicates that CpWRKY71 possesses transcriptional activation activity in yeast (Figure 3B).

### 2.3. Tissue-Specific Expression of CpWRKY71

**Figure 4:**
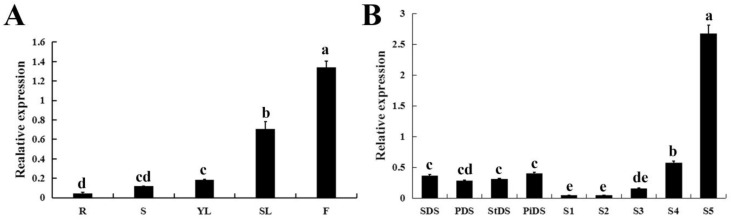
Expression patterns of *CpWRKY71* in wintersweet. (**A**) The expression pattern of *CpWRKY71* in different tissues of wintersweet. R: Roots; S: Stems; YL: Young leaves; SL: Senescing leaves; F: Flowers. (**B**) The expression pattern of *CpWRKY71* in different flower developmental stages of wintersweet. SDS, PDS, StDS and PiDS represent sepal, petal, pistil and stamen primordia the differentiation stage, respectively. S1–S5 represent the flower-bud, the petal-display, the initiating bloom, the bloom, the early-withering stage, respectively. *CpActin* and *CpTublin* were used as internal control. Data represent the mean of three biological repeats ± SD. Error bars indicate the standard deviation. Different lowercase letters indicate significant differences (*p* < 0.05).

qRT-PCR was performed to analyze the expression pattern of *CpWRKY71* in different tissues of wintersweet. The result shows that *CpWRKY71* was expressed in all the tissues examined, and the *CpWRKY71* transcript was most abundant in flowers. In vegetative organs, the expression level of *CpWRKY71* was much higher in senescing leaves than in other tissues (Figure 4A). Moreover, the expression pattern of *CpWRKY71* gene during flower development was also examined. The highest *CpWRKY71* expression was detected in the early-withering stage (S5), followed by flower primordia differentiation stages (SDS, PDS, StDS, and PiDS) and the bloom stage (S4), and the lowest level was present in the flower-bud (S1), the petal-display (S2) and the initiating bloom stage (S3) (Figure 4B).

**Figure 5:**
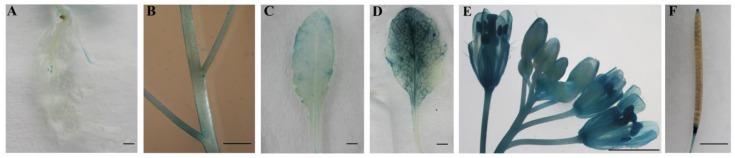
Tissue-specific GUS activity under the control of the *CpWRKY71* promoter. T3 homozygous lines were used for GUS staining analysis. (**A**) Roots, (**B**) stems, (**C**) young leaves, (**D**) senescing leaves, (**E**) the whole inflorescence, (**F**) siliques. Bars denote 2 mm.

To verify the tissue-specific expression pattern of *CpWRKY71* gene, a 3777 bp promoter fragment of *CpWRKY71* was fused to the *GUS* reporter gene to generate transgenic *Arabidopsis* plants (named *CpWRKY71pro*:*GUS*). Histochemical GUS staining showed that faint GUS activity was observed in the roots, stems and young leaves (Figure 5A–C) and strong GUS activity was observed in senescing leaves (Figure 5D). In inflorescence, flower buds showed weak GUS activity. However, GUS staining was increased with the flower blooming, and it was more pronounced in senescing flowers (Figure 5E). In the mature siliques, GUS staining was mainly detected in the abscission zone and the upper portion of the pods (Figure 5F). This result shows that the tissue expression pattern of *GUS* gene driven by *CpWRKY71* promoter in *Arabidopsis* was similar to that of *CpWRKY71* in wintersweet. Collectively, these results show that *CpWRKY71* was more highly expressed in senescing flowers and leaves than in other tissues.

### 2.4. The Expression Profiles of CpWRKY71 under Abiotic Stress and Hormone Treatments

**Figure 6:**
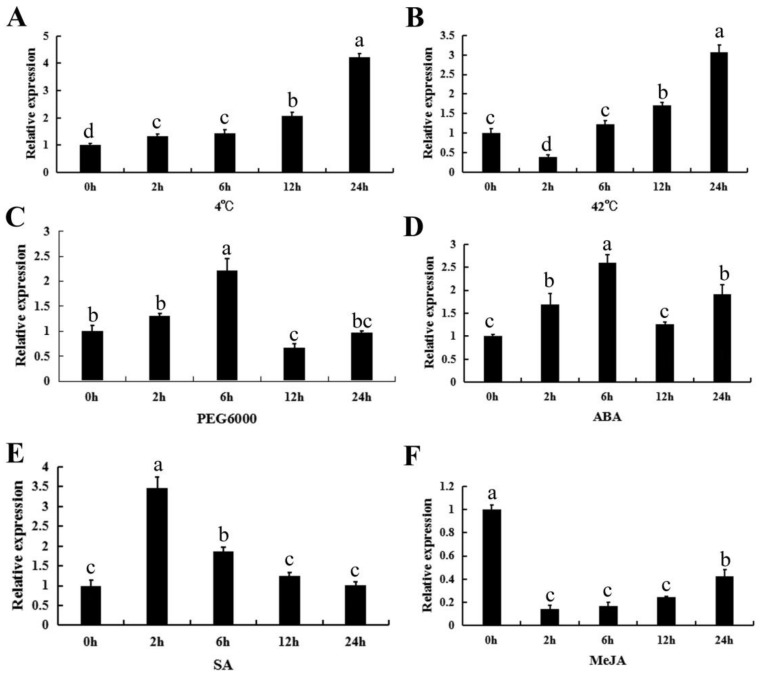
Expression patterns of *CpWRKY71* in response to abiotic stress and hormone treatments. Six-leaf stage wintersweet plants were exposed to treatment with (**A**) 4 °C, (**B**) 42 °C, (**C**) 15% PEG6000, (**D**) 50 µM ABA, (**E**) 2 mM SA, and (**F**) 100 µM MeJA. *CpActin* and *CpTublin* were used as internal control. The RNA was extracted from the top leaves at 0, 2, 6, 12 and 24 h post-treatment. Data represent mean of three biological repeats ± SD. Error bars indicate the standard deviation. Different lowercase letters indicate significant differences (*p* < 0.05).

To investigate the effect of stresses and exogenous hormones on *CpWRKY71* expression, the transcriptional levels of *CpWRKY71* under various abiotic stresses (drought, cold, and heat) and hormone treatments (abscisic acid, salicylic acid, and methyl jasmonate) were detected by qRT-PCR. These treatments were chosen according to the cis-acting elements present in the promoter region of *CpWRKY71* (Figure S1). For the cold treatment, the expression of *CpWRKY71* was gradually induced and reached a peak at 24 h (Figure 6A). For the heat treatment, *CpWRKY71* expression decreased at the 2 h time point, then gradually increased from 6 to 24 h and reached the peak at 24 h (Figure 6B). Under the PEG6000, ABA, and SA treatments, the expression level of *CpWRKY71* was induced and peaked at 6, 6, and 2 h respectively, then decreased (Figure 6C–E). For the MeJA treatment, the expression level of *CpWRKY71* was decreased dramatically after 2–12 h treatment and then showed a slight increase at 24 h (Figure 6F).

**Figure 7:**
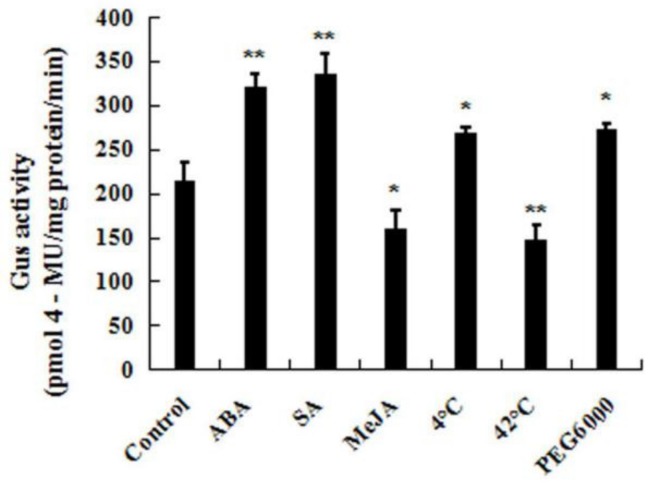
*CpWRKY71* promoter activity in response to abiotic stress and hormone treatments. Ten-day-old T3 homozygous *Arabidopsis* transgenic seedlings were treated with 50 µM ABA, 100 µM SA, 100 µM MeJA, cold stress at 4 °C and heat stress at 42 °C for 6 h, or 15% PEG6000 for 12 h. The plants grown under normal conditions were used as controls. Data represent the mean of three biological repeats ± SD. Error bars indicate standard deviation. Asterisks denote statistically significant differences compared with control, * *p* < 0.05, ** *p* < 0.01.

To further study the expression patterns of *CpWRKY71* under abiotic stresses and hormone treatments, the responses of *CpWRKY71* promoter to the above treatments were also examined (Figure 7). Ten-day-old homozygous *CpWRKY71pro*:*GUS* seedlings were used for the assay. The result shows that the GUS activities were significantly increased when seedlings were treated with cold, PEG6000, SA, and ABA. When the plants were treated with heat and MeJA, the GUS enzymatic activity was clearly decreased. Taken with our results, these data suggested that the expression of *CpWRKY71* was influenced by various abiotic stresses and hormones.

### 2.5. Heterologous Overexpression of CpWRKY71 in Arabidopsis Promoted Flowering Time and Leaf Senescence

**Figure 8:**
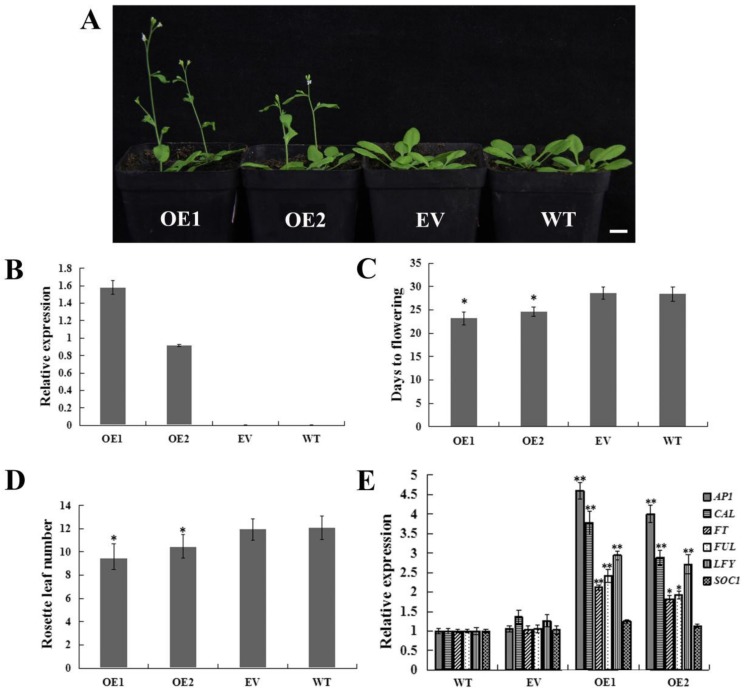
Flowering phenotype of *CpWRKY71* OE plants. (**A**) The flowering phenotype of 24-day-old plants under LD condition. Bar denotes 1 cm. (**B**) Transcription levels analysis of *CpWRKY71* in different lines. (**C**) and (**D**) The flowering time and rosette leaf number of plants grown under LD condition. Data represent the mean ± SD of 20 plants. (**E**) Expression levels of flowering-related genes in different lines. *AtActin* was used as an internal control. Data represent mean ± SD of three replicates. Error bars indicate standard deviation. Asterisks denote statistically significant differences compared with control, * *p* < 0.05, ** *p* < 0.01.

To further investigate the function of *CpWRKY71*, we transformed the *CpWRKY71* gene into *Arabidopsis*, and dozens of transgenic lines were obtained through hygromycin selection and PCR identification. The expression level was confirmed by qRT-PCR. Two homozygous T3 transgenic lines (OE1 and OE2) were selected for further phenotypic analysis (Figure 8B), the plants transformed with empty vector (EV) and wild type plants (WT) were used as control.

Under long day (LD) conditions, compared with EV and WT plants, OE1 and OE2 showed an early flowering phenotype (Figure 8A). OE1 and OE2 flowered at 23.2 and 24.6 days after germination (DAG) with 8.6 and 9.93 rosette leaves on average, respectively. While the EV and WT plants flowered at 28.4 and 28.6 DAG with 11.9 and 12.1 rosette leaves on average, respectively (Figure 8C,D). Thus, we examined the transcript levels of floral pathway integrators and the FMI genes *FT*, *LEAFY* (*LFY*), *SUPPRESSOR OF OVEREXPRESSION OF CONSTANS* (*SOC1*), *AP1*, *FRUITFULL* (*FUL*) and *CAULIFLOWER* (*CAL*) in OE, WT, and EV plants. The result shows that all these genes except *SOC1* were upregulated in OE1 and OE2 plants (Figure 8E). These results indicate that overexpression of *CpWRKY71* in *Arabidopsis* promoted flowering.

**Figure 9:**
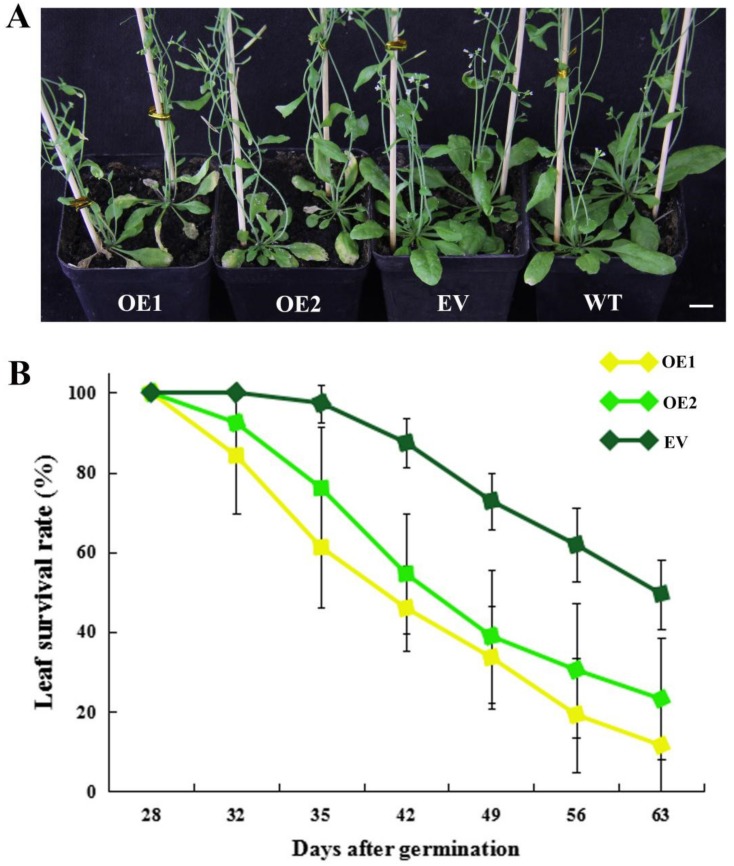
Precocious leaf senescence phenotype of *CpWRKY71* OE plants. (**A**) The leaf senescence phenotype of 35-day-old plants. Bar denotes 1 cm. (**B**) Leaf survival curves of OE and EV plants. Data represent the mean ± SD of 20 plants.

In addition to the early flowering phenotype, we also observed that *CpWRKY71* accelerated leaf senescence. OE1 and OE2 exhibited an early leaf senescence phenotype compared with the EV and WT plants (Figure 9A). The leaf survival rates of EV, OE1, and OE2 plants were counted from 28 DAG to 63 DAG. The survival curves further confirmed earlier leaf senescence in transgenic lines than in EV plants (Figure 9B). These data indicate that the constitutive expression of *CpWRKY71* accelerated leaf senescence in *Arabidopsis*.

## 3. Discussion

WRKY transcription factors are one of the largest transcription factor families in higher plants. To date, a large number of WRKY transcription factors have been identified in various plant species, such as *A. thaliana*, *Zea mays*, *Oryza sativa*, *Glycine max*, *Populus trichocarpa*, and *Vitis vinifera* [13,14,15,16,17,18]. Wintersweet is a superb winter flowering plant with economic importance in China. However, research about WRKY transcription factors of wintersweet has not yet been reported. In this study, a novel *WRKY* transcription factor gene, designated *CpWRKY71*, was isolated from wintersweet and functionally characterized in *Arabidopsis*. One WRKY domain and a C2H2 zinc finger motif were present in the CpWRKY71 sequence (Figure 1B), which is in accord with the characteristics of group II members. Phylogenetic analysis shows that CpWRKY71 is clustered with group IIc WRKY proteins and has a close relationship with AtWRKY71 (Figure 2). AtWRKY71 was reported to accelerate flowering via the direct activation of *FT* and *LFY* in *Arabidopsis* [25]. Other group members, such as *AtWRKY75* was reported to participate in the regulation of flowering in *Arabidopsis* [26]. In addition, *AtWRKY75* was also shown to act as a positive regulator of leaf senescence [27]. This will provide some clues for the functional analysis of *CpWRKY71* gene. CpWRKY71 is a nuclear-localized protein and has transcription activation activity in yeast, which was consistent with the results observed in previous reports [25,28], indicating that CpWRKY71 functions as a transcriptional factor (Figure 3).

The expression patterns of a gene can reflect its function to some extent. *PtrWRKY19* was highly expressed in stems, especially in the pith, further exploration revealed that *PtrWRKY19* participates in the regulation of pith secondary wall formation [29]. The expression of cotton *GhWRKY27* was induced by leaf senescence, further functional analysis showed that ectopic expression of *GhWRKY27* caused precocious leaf senescence phenotype in transgenic *Arabidopsis* [30]. In this study, the tissue-specific expression pattern of *CpWRKY71* showed that *CpWRKY71* expressed higher in flowers and senescing leaves than in other tissues (Figure 4A). During the flower development, high expression of *CpWRKY71* was detected in the early-withering stage and obvious expression of *CpWRKY71* was also observed in flower primordia differentiation and bloom stages (Figure 4B). These results indicate that *CpWRKY71* may play roles in wintersweet flower development and senescence.

Previous reports demonstrated that *WRKY* genes involved in the response to hormones and abiotic stresses [20,22]. In this study, the expression of *CpWRKY71* gene was affected by cold, heat, drought, ABA, SA and MeJA treatments (Figure 6). Corresponding with the expression patterns, abiotic stress and hormone-related cis-acting elements were found in the promoter sequence of *CpWRKY71 (*Figure S1). Compared with the promoter of *CpWRKY71*, cis-elements such as ABRE, TCA-element, CGTCA-motif, and TGACG-motif, were also found in the promoter sequence of *AtWRKY71.* However, different from that of *CpWRKY71*, cis-regulatory elements such as LTR, HSE and MBS were not observed in the *AtWRKY71* promoter *(*Figure S2). Therefore, the regulatory mechanism of *CpWRKY71* in wintersweet might be different from that of *AtWRKY71* in *Arabidopsis* in some respects, even though *CpWRKY71* shares some similarity with *AtWRKY71*.

WRKY proteins have been reported to be involved in various developmental processes, such as trichrome and seed coat development [31], dormancy [19], senescence [20,27], flowering [9,21,25,26], and stress responses [23]. In this study, overexpression of *CpWRKY71* in *Arabidopsis* caused an early flowering phenotype. The expression levels of flowering integrator and FMI genes (*FT*, *LFY*, *AP1*, *CAL,* and *FUL*) were increased in transgenic lines (Figure 8). The early flowering phenotype and upregulation of flowering-related genes exhibited some similarities to its orthologue *AtWRKY71*, this indicates that *CpWRKY71* participated in the regulation of flowering time in *Arabidopsis*. According to several previous studies, different WRKY proteins may be involved in the regulation of flowering in distinct ways. For example, *Arabidopsis* WRKY12 and WRKY13 were reported to oppositely modulate flowering under SD conditions via directly regulating *FUL* [32]. AtWRKY71 was found to promote flowering by directly activating the expression of *FT* and *LFY* [25]. AtWRKY75 was reported to positively regulate flowering via interaction with DELLA proteins [26]. These results, especially that of *AtWRKY71*, provide the foundation for further analyzing the role of *CpWRKY71* in the control of flowering time. Many *WRKY* genes have multiple functions in plants [26,27,33,34,35,36]. For example, *Miscanthus MlWRKY12* was reported to participate in pith secondary cell wall formation and the regulation of flowering [9]. Rice *OsWRKY78* was found to be involved in seed development and stem elongation [37]. In this study, *CpWRKY71* overexpression plants showed precocious leaf senescence in addition to the early flowering phenotype (Figure 9), which is in accordance with its expression pattern. Previous studies have shown that some *WRKY* genes participated in the regulation of leaf senescence. For example, *Arabidopsis* WRKY54 and WRKY70 were reported to co-operate as negative regulators of leaf senescence [38]. *Arabidopsis* AtWRKY45 was shown to participate in the regulation of leaf senescence through interaction with DELLA Protein RGL1 [39]. Leaf senescence is influenced by environmental and endogenous cues, such as abiotic stresses and hormones [30]. For instance, *Arabidopsis JUB1*, an H_2_O_2_-induced NAC transcription factor gene, overexpression of *JUB1* in *Arabidopsis* resulted in delayed senescence through lowering the cellular H_2_O_2_ level [40]. *Arabidopsis S3H* which was induced by SA, the *S3H* gene knockout mutant *s3h* displayed early leaf senescence phenotype, and *S3H* regulates leaf longevity by mediating SA catabolism [41]. The *foxtail millet SiNAC1* gene expression was induced by ABA and senescence. Functional analysis revealed that *SiNAC1* regulates leaf senescence through the ABA pathway [42]. In this study, *CpWRKY71* expression was influenced by various stresses and hormones (Figure 6). Therefore, we speculate that *CpWRKY71* might affect leaf senescence through regulation of one specific or multiple pathways. In the past decades, many *WRKY* genes have been reported to affect both flowering and leaf senescence [20,25,26,32]. Taken together, our results indicate that *CpWRKY71* may function in regulating flowering time and leaf senescence in transgenic *Arabidopsis*.

So far, research about WRKY transcription factors of wintersweet has not yet been reported. Hence, our study laid a good foundation for further analysis of *WRKY* genes in wintersweet. Meanwhile, the functional characterization of *CpWRKY71* broadens our knowledge of the roles that *WRKY* genes may play in woody plants. Moreover, these findings also enrich our knowledge of the molecular mechanism about flowering and senescence in wintersweet.

## 4. Materials and Methods

### 4.1. Plant Materials and Growth Conditions

Seeds of wintersweet used in this research were collected from Southwest University. The seeds were treated with 95–98% sulfuric acid for 30 min. After that, wintersweet seeds were cleaned under running water and then sown into pots filled with a peat and perlite mix (1:1) under greenhouse conditions (16 h light/8 h dark photoperiod, light intensity of 20000 lx, 25 °C).

For the tissue-specific expression pattern of *CpWRKY71*, the roots, stems, and young leaves were collected from six-leaf stage plants, senescence leaves and flowers at stage 4 (bloom period) were collected from the adult wintersweet plant [1]. Three biological replicates of each tissue were collected.

For the expression pattern of *CpWRKY71* gene in different flower development stages, samples at different flower developmental stages were collected as previously described [5]. Three biological replicates of each sample were collected.

Seeds of the *A. thaliana* ecotype Columbia were sown on solid Murashige and Skoog (MS) medium before vernalization at 4 °C for three days and then grown in a growth chamber at 22 °C with the16 h light/8 h dark photoperiod (120 µmol photons·m^−2^·s^−1^).

### 4.2. Gene Cloning

Total RNA isolation and the first-strand cDNA synthesis were performed as described by Liu et al. [5]. Total genomic DNA was extracted from the wintersweet leaf using CTAB method [43]. Specific primers *CpWRKY71*-F/R (Table S1) were designed to clone the cDNA and DNA sequence of *CpWRKY71*. The PCR products were isolated from the gel using the Agarose Gel DNA extraction kit (Tiangen, Beijing, China), then cloned into the pMD19-T vector (TakaRa, Dalian, China) and sequenced by TsingKe Company (TsingKe, Chengdu, China). The multiple alignments were performed using BioEdit software. MEGA 5.0 software with the NJ method (1000 BootStrap replicates) was used to construct the phylogenetic tree of CpWRKY71 and WRKY proteins from other species.

The 5’-upstream sequence of *CpWRKY71* was isolated according to the protocol of the Universal Genome Walker Kit (Clontech, USA). Specific primers GSP1 and GSP2 (Table S1) were designed according to the *CpWRKY71* gene sequence to clone the promoter. The purification and sequencing of the PCR products were performed as described above. The putative cis-acting regulatory elements were searched with the PLACE database (http://www.dna.affrc.go.jp/PLACE) and PlantCARE (http://bioinformatics.psb.ugent.be/webtools/plantcare/html/) [44].

### 4.3. Plasmid Constructs

For the subcellular localization assay, the open reading frame (ORF) of *CpWRKY71* without termination codon was cloned into the modified pCAMBIA 1300 vector [5] via SacI and SalI sites, to construct the plasmid *35S*:*CpWRKY71*-*GFP*. For transcriptional activity analysis, the ORF of *CpWRKY71* was inserted into pGBKT7 via XmaI and Sal I sites, to create the plasmid pGBKT7-*CpWRKY71*. For GUS histological assay, *CpWRKY71* promoter region was cloned into the vector pBI121 to replace the CaMV35S promoter via HindIII and BamHI sites, to construct the plasmid *CpWRKY71pro*:*GUS*. The plasmid *35S*:*CpWRKY71*-*GFP* was also used to generate *CpWRKY71* overexpression plants. The primers are listed in Table S1.

### 4.4. Subcellular Localization and Transactivation Activity Assay of CpWRKY71

The recombinant plasmid *35S*:*CpWRKY71*-*GFP* and the empty vector *35S*:*GFP* were separately introduced into *A. tumefaciens* strain GV3101. The young leaves of tobacco (*N. benthamiana*) plants were infiltrated with *A. tumefaciens* cells harboring *35S*:*GFP* and *35S*:*CpWRKY71*-*GFP*, respectively. Confocal laser microscopy (Olympus, Japan) was used to observe the GFP fluorescence.

The recombinant plasmid pGBKT7-*CpWRKY71* and pGBKT7 were transformed into the AH109 yeast strain, respectively. SD/-Trp medium was used to select the positive transformants. SD/-His (containing 10 mM 3-AT) and SD/-His/X-α-gal medium (containing 10 mM 3-AT) were used for transactivation analysis. The pGBKT7 empty vector was used as a negative control.

### 4.5. Plant Treatment

In order to understand gene expression patterns under different abiotic and hormone treatments, wintersweet plants at the six-leaf stage were used [45]. For the drought treatments, plants were irrigated with 15% PEG6000 [46]. For the cold and heat treatments, plants were treated with 4 °C or 42 °C [45], respectively. For exogenous hormone treatments, wintersweet plants were sprayed with 50 µM ABA, 2 mM SA, or 100 µM MeJA [45,47], respectively. Two top leaves of one individual wintersweet plant were collected as one replicate. There were three biological replicates for each treatment. The samples were collected at 0, 2, 6, 12, and 24 h after treatment, frozen in liquid nitrogen and stored at −80 °C ultra- freezer until used.

To understand the effects of different treatments on *GUS* gene driven by the *CpWRKY71* promoter in *Arabidopsis*, ten-day-old homozygous *Arabidopsis* T3 seedlings were used [48]. For drought stress, seedlings were transferred into a new plate, then irrigated with 15% PEG6000 for 12 h [49]. For the cold and heat treatments, seedlings were treated with 4 °C or 42 °C for 6 h [48]. For exogenous hormone treatments, *Arabidopsis* seedlings were sprayed with 50 µM ABA, 100 µM SA, or 100 µM MeJA for 6 h [50,51,52], respectively. The seedlings grown under normal conditions were used as controls. Each experiment contained three biological replicates. These samples were then used for GUS activity analysis.

### 4.6. Quantitative Real Time-PCR Analysis

qRT-PCR was performed on a Bio-Rad CFX96 Real-time system machine using the Ssofast EvaGreen Supermix (Bio-Rad, Hercules, CA, USA). The PCR procedure was as follows: 95 °C for 30 s, followed by 40 cycles of 95 °C for 5 s, 60 °C for 5 s and 72 °C for 5 s, and a melt cycle from 65 to 95 °C. *CpActin*, *CpTubulin,* and *AtActin* were used as the internal reference for the gene expression analysis in wintersweet and *Arabidopsis*, respectively [5]. The comparative CT method was used to analyze real-time PCR data [5]. All the primers used for qRT-PCR are listed in Table S1.

### 4.7. Arabidopsis Transformation

For GUS histological assays, the recombinant plasmid *CpWRKY71pro*:*GUS* was introduced into *A. tumefaciens* strain GV3101 and then transformed into *Arabidopsis* via the floral dipping transformation method [53]. T0 seeds were sowed on MS media containing 50 µg/mL kanamycin. *CpWRKY71* overexpression plants were generated as described above. The positive plants were selected on MS media containing 25 µg/mL hygromycin. The seedlings with the resistance grown in a growth chamber under long-day conditions (22 °C with the 16 h light/8 h dark photoperiod and 120 µmol photons·m^−2^·s^−1^ light intensity).

### 4.8. GUS Histochemical and GUS Activity Assays

Histochemical staining was performed according to the method described by Khan et al. [50]. Samples were immersed into GUS staining solution. After incubation in the dark at 37 °C overnight, they were rinsed with 70% (v/v) ethanol to completely remove chlorophyll and then photographed by stereoscopic microscope (Nikon, Japan).

The procedure of the GUS fluorometric assay was performed as described by Niu et al. [49]. The protein concentration of the samples was measured according to the Bradford method (Bradford, 1976) by using Modified Bradford Protein Assay Kit (Sangon, Shanghai, China). 4-Methylumbelliferyl-β-d-glucuronic acid (MUG, Sangon, Shanghai, China) was used as the substrate. The GUS activity was measured using a Varioskan Flash Spectral Scanning Multimode Reader (Thermo Fisher, Waltham, MA, USA) with 365 nm excitation and 455 nm emission. GUS enzyme activity was expressed as pmol 4-MU produced per mg protein per minute.

### 4.9. Phenotype Analysis

The flowering time was measured as described by Zhang et al. [26]. The day when the first visible flower appeared, and the total rosette leaf number were measured to determine the flowering time. The completely yellowed leaf was regarded as a dead leaf [41].

### 4.10. Statistical Analysis

Statistical analysis was conducted using SPSS 17.0 software (SPSS, Chicago, IL, USA). The significance of differences was analyzed by Student’s *t*-test. It was considered significant with a *p*-value of 0.05, or very significant with a *p*-value of 0.01.

## Supplementary Materials

Supplementary materials can be found at https://www.mdpi.com/1422-0067/20/21/5325/s1.

## Abbreviations

ABA	Abscisic acid
CTAB	Cetyltrimethylammonium Bromide
GUS	β-glucuronidase
MeJA	methyl jasmonate
PEG6000	Polyethylene glycol 6000
SA	Salicylic acid
qRT-PCR	Quantitative Real Time-PCR
