# Supplementary material for: CpWRKY71, a WRKY Transcription Factor Gene of Wintersweet (Chimonanthus praecox), Promotes Flowering and Leaf Senescence in Arabidopsis
Source: Int J Mol Sci. 2019 Oct 25;20(21):5325. doi: 10.3390/ijms20215325 (PMC6862124; doi:10.3390/ijms20215325)
Supplement: Supplementary file 1 [file ijms-20-05325-s001.pdf]

International Journal of *Molecular Sciences*

MDPI

Table S1. List of primers.

Primer name	Primer sequence (5′-3′)
CpWRKY71-F	AAGCTAAACCTCTTCCCTCT
CpWRKY71-R	CCACACTAGGATGTTGGTTCATG
GSP1	GCCAACACTCCAGAAGCACTCCC
GSP2	CATCGCCAGAGCCGAACACG
CpWRKY71-1300-F	GCGAGCTCATGTCTGAAAATAGAGATTCC
CpWRKY71-1300-R	GCGTCGACTGGTTGTCTAGGGATGAAAG
CpWRKY71-pGBKT7-F	TCCCCCCGGGGATGTCTGAAAATAGAGATTCCAGCC
CpWRKY71-pGBKT7-R	GCGTCGACTGGTTGTCTAGGGATGAAAGAAGG
pCpWRKY71- PBI121-F	CCCAAGCTTTTTGAAATAAACCAGTATGATATGCTT
pCpWRKY71-PBI121-R	CGCGGATCCTCTGGTGCGGGAGAGAGGAGC
CpActin-F	AGGCTAAGATTCAAGACAAGG
CpActin-R	TTGGTCGCAGCTGATTGCTGTG
CpTublin-F	GTGCATCTCTATCCACATCG
CpTublin-R	CAAGCTTCCTTATGCGATCC
AtActin-F	CTTCGTCTTCCACTTCAG
AtActin-R	ATCATACCAGTCTCAACAC
RT-CpWRKY71-F	TGACCACTACCACGATGACAACA
RT-CpWRKY71-R	GGATTGCCACTGGGTCCTCTA
RT-FT-F	TTCCAAGTCCTAGCAACCCTCACC
RT-FT-R	TTCTTCCTCCGCAGCCACTCTC
RT-FUL-F	GCCTCAATACTGCGTAACCTCC
RT-FUL-R	GGTAGGACGTAACATCCAAGCC
RT-CAL-F	AAGAAGACCAAACGGCGATG
RT-CAL-R	GGCGTAACAGCCAAGGTAATTG
RT-LFY-F	CTCTATTTGGTATGTTCCAACAAAG
RT-LFY-R	CTAATACCGCCAACTAAAGCC
RT-SOC1-F	AGCTGCAGAAAACGAGAAGC
RT-SOC1-R	TGAAGAACAAGGTAACCCAATG
RT-AP1-F	CATGGGTGGTCTGTATCAAGAAGAT
RT-AP1-R	CATGCGGCGAAGCAGCCAAGGTT

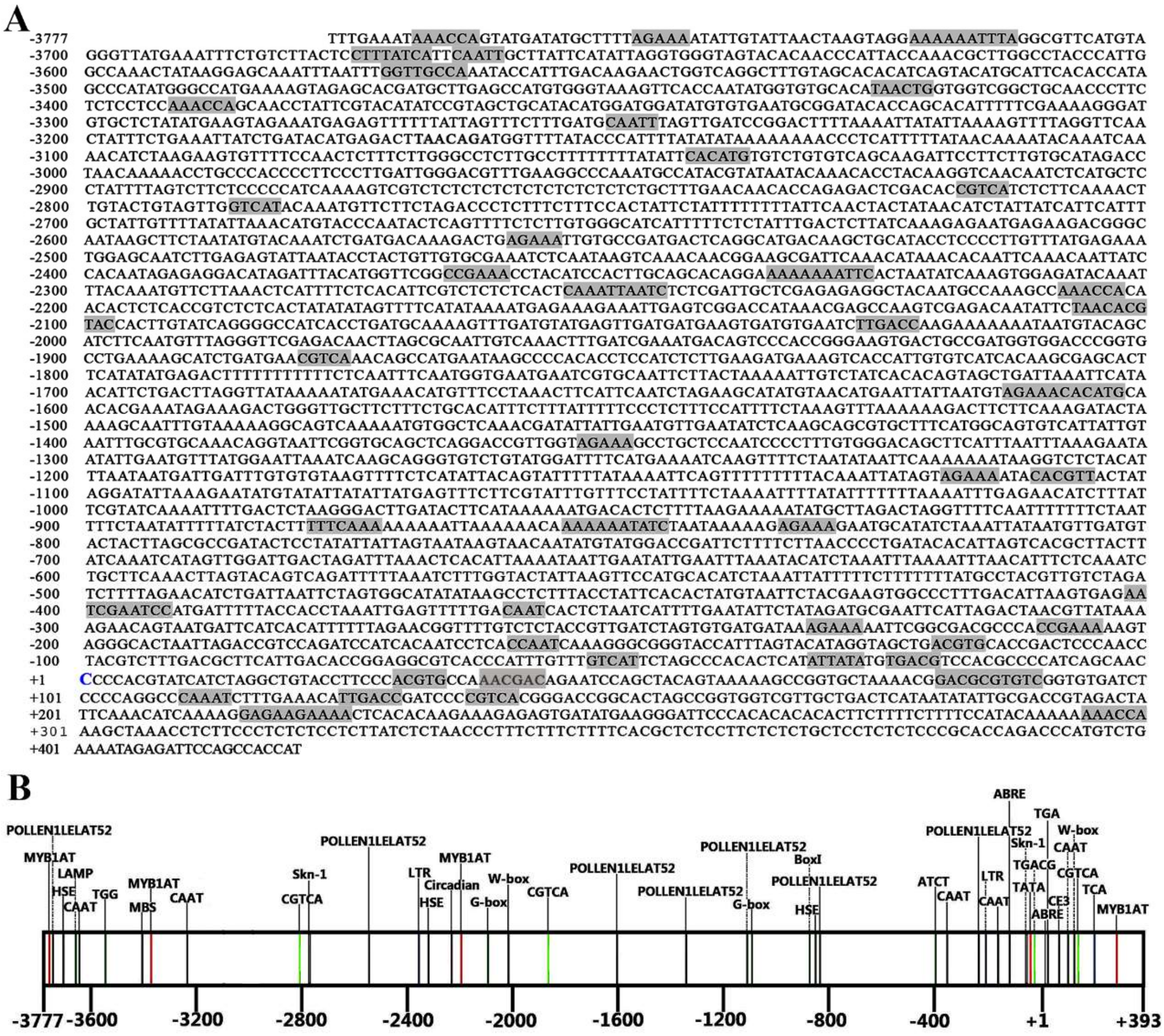

Figure S1. The DNA sequence analysis of *CpWRKY71* promoter. (**A**) The nucleotide sequence and putative cis-acting regulatory elements predicted in the promoter region. The putative cis-acting regulatory elements are marked by the grey shades. The putative transcriptional start site is designated as +1 and marked in blue. The translation start site (ATG) is marked by red box. (**B**) A diagram of the promoter region of *CpWRKY71* with potential cis-elements binding sites.

A

-4136 TCATATAACTTATGATTAGTAGTACGTCGTTCGAAT
-4100 ATAGATCAGTCATTATCATTAGATAAGCCATCTTTCTACAAAAATAAAGTGGCTATATTGAAAAAGTAATTAAAAAGACAGTTGAACAACATTTTATGCA
-4000 AAGTGTGTTACGATCCGTACAATAATCAATTAATAAAACGCTAAGACTTGGTCTTCTTAAATACTAGTGATTAGAAAACGACATTAGTGGAGTGGATGTT
-3900 GAACTTGAATTCTCGAATTCAATTGAGCCTAAACGTTGACTTTTGACTTTTCCAGTCATTGGTTTCCTTTTCGATTTCCTAATACTTTTTTTGTGTCCC
-3800 CTTGTCTCTAGTCTTTGTTGTATTCATAATTATCTCATATAGTAAAAATGAATGTAACACTACTCGATAATGCATCTACTTTTTTTTTAATTGATTATAT
-3700 GTCTATATTAGAAGATGGAACATTTCGGATTTTTAAATATCATTTTCGGAAACATAAAGCTTAACACTTTTGGGAACAAAATTTCCGAACTTTTGAGAAT
-3600 AGCTAATTGCCGTCGATGGTTACAAGAGTGGAGAAACGTGAGATAGGTGGACCTGAACGGTCAGGGCATTATGTAAGTGATTTTTCATTGTTTTGACGAA
-3500 ATTATGGATAAGTAAACATTACGATTATGAAAATTAATCATATCTCAGAGTTGTCATTTACCTATTGTACATAAATATTACCTAACGAAATACCTGCTCT
-3400 AAACGTCGAAACATTCATTATTTTAGTGTCACACTCAACTAGAAAATAGAATTGACATGAGTAATGAGTTTTTCAAAGTCATATACTTTTCATGTTTTTG
-3300 TTATATAATTTAAATTTGGACTAGAACTGAATACTTCTAAGTTTTGACGATAGAGCATTACATCTACTTGTGAAACTAAATGTATCACACGACATGTAGA
-3200 TGATTGAAAAAGAAGAGAAATTATGATTCATAAGAAACATTTATTTAGCAAAAAAACAATTATAATATGTTCTTTTCCCCCTAAGTAAAGAGAATTATTA
-3100 GCTTAAACTATTTTGGTACTGAGAGTGAAGGATTTGAAAAAGGTTATTGAAATATTAGGTTATCTATGCTATTTTTAACTAGCAAATATCGAAATACTTT
-3000 TATTATATTATATTTGTCGTCAAATCGACAAAATTAAAACTAGCAAAATTGTAGGTGAAATGTCAAAGAGAAAGTGACAACCGTGCACCTTTTTTACTTG
-2900 TACAAGTGTCTCCACAATTTACCTATCAATTAGCGTCTCGTAGTATTTTTGAATTTTAAACTATGATAAGTTTTTCCCATTAAATTCAAAGTTTTCTACA
-2800 AATAATTTCTATTTATTTTATAATAGTAAACTCTTAAACGTCTAATTGACTTATCAAACACCACTAGACGTTAGGTGTACTAGAAATGCCACACAAAACT
-2700 TCGATTAGTGCTTAGCATTTTTGAACTTCCTCCATGAAAAAACGAAAAGCGAATATAATAAGAAGGTTTTTTGGCTCCCTAATTTCCAAGAAATAGTGTT
-2600 GTTGAACTGTACTCACTTGAACTCTTTTGGTAAAAGAATTATGGTTTAAGCCTTTAAGGTTACTTCTTCAATTGTTATACTTACAAAAACACGCAAACGA
-2500 ATATGGTTAGTGGGTTCGACCTCTCATCACATAGTGATGAAATCCGTTAAAAAATTAAAGAAACCATAAACCAAAGTCACCAAACACTCAAAATTCTAAA
-2400 ATTACAAAGCAAATACATAAAATGACATAGGGCTTGGGTCATCAAAGACAGGGGGAATGAAGAGCACGTGATATGCAGTCTCATGCACGACTTTGCTGAT
-2300 CCTAATGGTCAAATACGGTGTCATTTGCTTCCACGTCTCTTAATTACATTTTATGTTTCTAACACTAAAAAGTAAGAATGCAATCATATGGTTTAAAGCC
-2200 GTGTACGTACAGACAAATTGCTCGAAATAGCCAAACTCGATTCACTCGTAATATATATGTTTTTCCAAGTTCAAACAATGGTTAGATAATAGAAATGTTT
-2100 AGATCCAATCATTGTGTTTTTATTTTTGTGAGTATATTTATTCTTAAGATTCTTTTTCGACGTCTAGAGATATGGTTATTTGTTAGTGTCTAGACTTATC
-2000 TAATCTTAAATAGAAAAGCTAAAGAAAGTGAAGTCATTGCTTTGAAAGACGTTGACTGTTCACAAAAACAACTAGTCTTCGTTGACTTTGAATCAACAAC
-1900 AATAATGTAGCTCCAAAGAAAGGAGTATATCTAATTCAAACCCTTATTTAAACAAGAAAGAAAGAATTCGTAAGCTTTTCTATGGAGGTTATTTGTATTA
-1800 ATGAACCAAAAAGTTATATAAAAAGGGTTATAGAGAGAGGCGCGCAAAATGAAGACTTCTGTTAACTATTCTTTGGCTTCTTGTATATGCGTATTAATAT
-1700 ATGATTCGTTATAATTCACCAAGAAAGATTTCTTTTGACGAGTAGAAAGAGTTGGAAGTATATGCATAATGTCTACATAACTTTATACATTTGACCTCTA
-1600 TTTTATCTTTATTTTTAATATTCCTCGGTAGGACTTCTACGCACATAAACTTTTAAAACCTAATCAGATTGCATTTATATATACCTCAATTTCTGATATT
-1500 GTCTATGGACATGGTGACAGTTTAAATTTGTTATGTACTACATGATATGGTGTTATGGTGATGATCATGTCCACTATGAGATATCTACGAAGATATATGT
-1400 ACGTATGTTTTCCAACATATTTTCTATTTTATTTTTACGTTTAGTCAAGACCATATTCATAGCGTCAATCTCCATGTGAATTCTAATAATGTAGACTCTT
-1300 AAGAATAAACTATTCATTGTCTTAACCAAATGTATGTTTACAAGACGAAAAAAACACAAGGAAAGAAAAAGGAAAGAATGAAAGCCAAAACATAGTGTGG
-1200 TAGATAGGATTAGAGAGAAATATATAGTTAAATGCTCCTTTATAGAAACCACTTTTAGTTCAATAGCTCTCTAGCTATATTTAATTTTTAGTTGAAATTG
-1100 TCAAACCTTTCTTTTTCATATACATCAAAATATTTTAACAATTTATACCCATTAAAGTCTTCTAATAAGATTATGACCCTTCCAAACTTTTCAGTATCTC
-1000 CCTCTTGAATTCTTTCAAATTAGTTTAGTATTTCACATTTTAGAGAGACTATAATCTCAAATTACATACCATAGTTGATACTTTGGTTCGACAAGTAATT
-900 TAATAATATTAAGAACGATTGTTGCTGGTAGCTAGTAAATTGGTCATATCTATTTGGTTGATAGATCATATATTAAAATTTGATCTCAGATCATCGACCA
-800 TCATACACATAATTTGTAGTATAATTCATCTTTTATGTCCATTTTCTGCCATTGCGGGTTACTCAAATAAAGTTGGGAATTAATTGCAAATTTTTTCCTC
-700 TAATACAATTAGAAACGATGACAAAAAAAAAGGTATATATATAATTTCTAACAACGGAGCATACGCCTAAAATATCCAAAATATGCTTCATGAACCTTAA
-600 TATAATTAAGGGTAACGAAAAACAGTTTCGATTGGAGGGTCAAATAGTGTTTCCAAGATTTAACTCCATAAATCAACATCCAGTTTGGGTAAAATAATTT
-500 TGTCTTGTGTAAAAATTTTCCTAGCTAAAACATTAAATCACACTGAAGTAGACTATCTTGGGATTATTTTTTAGTTTTTTGATAACCACTACATTGATAT
-400 TTTCAAATTGAATATACAAAGGACAGTCTTCTACATATGGTTTGAATGCAAATCTATGAATCTTCATATGGTAGGTATATTATACCTCAATAAAAGATAT
-300 TGATCTTAAACTGATATGTTAGAAATCCAACGTTATAAAGTACAGGACGTATAATTTGGTACATTAAAAGAGATTAGTTACTCAATTTATAACCAATCGG
-200 AAATATGGTATCATAATTCCATTGATTTTACTGACCAAACATTACCAATGGACATTTTTATTTTTTCCAAGTGATATCGCTCCAAAATGATTTAATTAAG
-100 ACCTTAATAGAATATTCAGAACAAAAAACCATCCAAGTATTAAAGTAAAGTTGAATGGACTTGTATTTATAAGACTTGATCACCCAAAGTCCACATATAA
+1 CCTAACACCTAAGTTTGAAGAACCAAATCTCTTCCTCTTCTTCTCTTTCTCCTCTTCTTCA

B

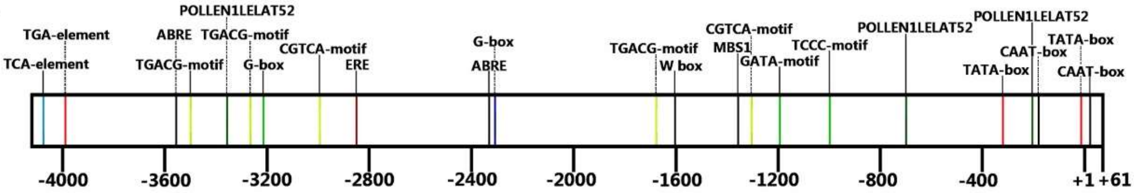

Figure S2. The DNA sequence analysis of *AtWRKY71* promoter. (**A**) The nucleotide sequence and putative cis-acting regulatory elements predicted in the promoter region. The putative cis-acting regulatory elements are marked by the grey shades. The putative transcriptional start site is designated as +1 and marked in blue. (**B**) A diagram of the promoter region of *AtWRKY71* with potential cis-elements binding sites.
